# A Market-Basket Approach to Predict the Acute Aquatic Toxicity of Munitions and Energetic Materials

**DOI:** 10.1007/s00128-016-1800-0

**Published:** 2016-04-18

**Authors:** Lyle D. Burgoon

**Affiliations:** US Army Engineer Research and Development Center, 3909 Halls Ferry Road, Vicksburg, MS 39180 USA

**Keywords:** Computational toxicology, Quantitative structure activity relationship, Aquatic toxicology, QSAR, Predictive toxicology, Munitions, Energetics, Fish toxicity, Acute toxicity

## Abstract

An ongoing challenge in chemical production, including the production of insensitive munitions and energetics, is the ability to make predictions about potential environmental hazards early in the process. To address this challenge, a quantitative structure activity relationship model was developed to predict acute fathead minnow toxicity of insensitive munitions and energetic materials. Computational predictive toxicology models like this one may be used to identify and prioritize environmentally safer materials early in their development. The developed model is based on the Apriori market-basket/frequent itemset mining approach to identify probabilistic prediction rules using chemical atom-pairs and the lethality data for 57 compounds from a fathead minnow acute toxicity assay. Lethality data were discretized into four categories based on the Globally Harmonized System of Classification and Labelling of Chemicals. Apriori identified toxicophores for categories two and three. The model classified 32 of the 57 compounds correctly, with a fivefold cross-validation classification rate of 74 %. A structure-based surrogate approach classified the remaining 25 chemicals correctly at 48 %. This result is unsurprising as these 25 chemicals were fairly unique within the larger set.

The US Army develops novel munitions and energetic materials for the US Department of Defense (DoD) in order to fulfill DoD national security missions. As part of its environmental quality and occupational health responsibilities, the US Army requires knowledge of what chemicals may be hazardous to human health and the environment, under what exposure circumstances, and how to manage potential risk.

Predictive toxicology can help the US Army achieve its environmental quality and occupational health responsibilities by identifying potential safer alternatives to currently used chemicals, or to identify a small number of potentially less toxic leads amongst a large number of new molecular entities. This is similar to the challenges faced in the pharmaceutical industry where toxicity remains a major source of attrition late in pharmaceutical development (McKim 2010).

One of the challenges being faced by the US Army is that the novel energetics and munitions currently being developed are typically outside of the chemical space of commercial off-the-shelf quantitative structure activity relationship (QSAR) programs, such as TOPKAT. This means that new data and models need to be built to meet the Army’s predictive toxicology needs.

Once the DoD begins using munitions and energetics they will be released into the environment. The potential safety/risks associated with these chemicals once they enter the aquatic environment is important, as we need to minimize potential adverse impacts on a myriad of species, including small fish. Thus, being able to accurately predict potential chemistries (i.e., toxicophores) that are associated with specific ranges of potential small fish toxicity is important. If we are able to avoid certain toxicophores, or drive towards the development of chemicals with safer toxicophores, then we may be able to manage potential aquatic harm before the chemicals are ever developed. This will ultimately save money and time developing compounds that are later found to be too environmentally toxic for use.

This paper describes a method for identifying potential toxicophores for munitions and energetics of US Army interest that predict categories of small fish toxicity. As more data is obtained over time, we will be able to update the database and update the predictive toxicophores.

##  Materials and Methods

The US Army had previously built a database of 96-h fathead minnow LC50 data for 57 chemicals by examining the literature or running its own in-house testing program. This database was used to build the toxicophore model. The full details of the analysis, the code, and all of the data are available on GitHub (https://github.com/DataSciBurgoon/toxicophore).

**Fig. 1:**
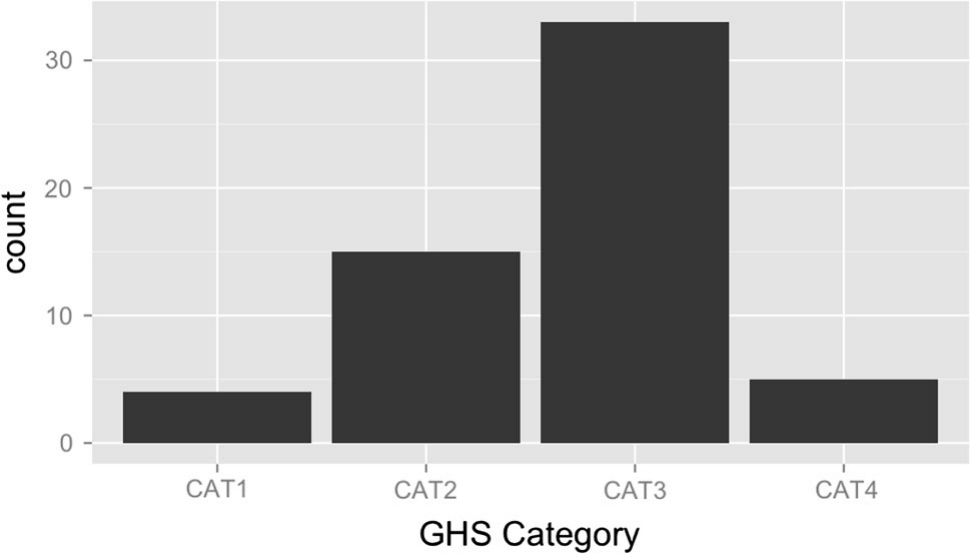
Number of chemicals represented in each GHS category. CAT3 and CAT2 are the largest categories, respectively, followed by CAT4 and CAT1. The algorithm had difficulty identifying predictive rules for CAT4 and CAT1 due to the small number of chemicals in each category

The United Nations Globally Harmonized System of Classification and Labeling of Chemicals (GHS)(United Nations 2013) has three categories for acute aquatic toxicity: Category 1 (CAT1): 96 h (acute) fish LC50: <1 mg/L; Category 2 (CAT2): 96 h (acute) fish LC50: 1 mg/L < x ≤ 10 mg/L; Category 3 (CAT3): 96 h (acute) fish LC50: 10 mg/L < x ≤ 100 mg/L. A 4th category was also created (CAT4): those chemicals whose 96 h (acute) fish LC50 > 100 mg/L. CAT3 has the most chemicals, followed by CAT2, CAT4 and finally CAT1 (Fig. 1). In fact, the number of chemicals in CAT1 and CAT4 is so small that it is difficult to generate rules.

The ChemmineR package (Cao et al. 2008) was loaded into R (v. 3.2.2) along with the arules package (Hahsler et al. 2005). Open Babel (O’Boyle et al. 2011) was used to convert a file containing SMILES codes for all 57 chemicals into an sdf file using the bash command line. Next, ChemmineR was used to fingerprint the chemicals using the 4096 most frequently occurring atom pairs from the Drugbank database.

Probabilistic rules were identified using the market-based analysis/frequent itemset mining algorithm, Apriori (Borgelt 2012). Market-basket analysis is typically concerned with identifying purchased products in a grocery store that predict other items an individual may purchase. For instance, a rule may predict that individuals who buy breakfast cereal and oatmeal may be more likely to also purchase milk in the same transaction. This rule would be written as “breakfast cereal AND oatmeal => milk”, where the => represents the word “then”.

Similarly, if one considers atom-pairs within a chemical and the lethality data for fathead minnows to be all of the products within a supermarket, then the goal is to find rules that capture those atom-pairs that are predictive of the GHS category. Thus, the rule would look similar to “atom-pair X AND atom-pair Y => GHS category”.

The Apriori algorithm also calculates several statistics that help with rule interpretation. The confidence in the rule is the same as the conditional probability. So in the example above, the confidence is equivalent to P (Milk| Breakfast Cereal, Oatmeal). For this application, the confidence, or conditional probability of the rule, is the most important statistic, as it tells us the probability that a chemical will be within a given toxicity range given the structural characteristics in the rule.

The chemical fingerprints were filtered to only keep those atom-pairs that exist within the dataset (i.e., those where at least one chemical has the atom-pair), and that have a confidence of at least 75 %. This resulted in 3390 rules. The four hazard categories from the dataset were appended to the filtered fingerprint object in R. Note that these four hazard categories are the original 3 from the UN GHS system, as well as a 4th category to capture those chemicals with an LC50 > 100 mg/L. This matrix was then used in the apriori algorithm to generate rules. These rules were further examined to identify structural characteristics that are associated with acute aquatic toxicity. The prediction accuracy of the apriori rules was assessed using fivefold cross-validation.

The surrogate approach uses the GHS classification of the most structurally similar/nearest neighbor chemical. The nearest chemical based on structural similarity was identified using Tanimoto distance.

A more quantitative approach was ruled out as the goal of this project was simply to predict the GHS categories. In addition, due to the small size of the dataset, the ability to make highly quantitative LD50 predictions would likely have resulted in such large uncertainty, that in the end, the approach would have ended up appearing more like the semi-quantitative approach taken here.

## Results and Discussion

**Fig. 2:**
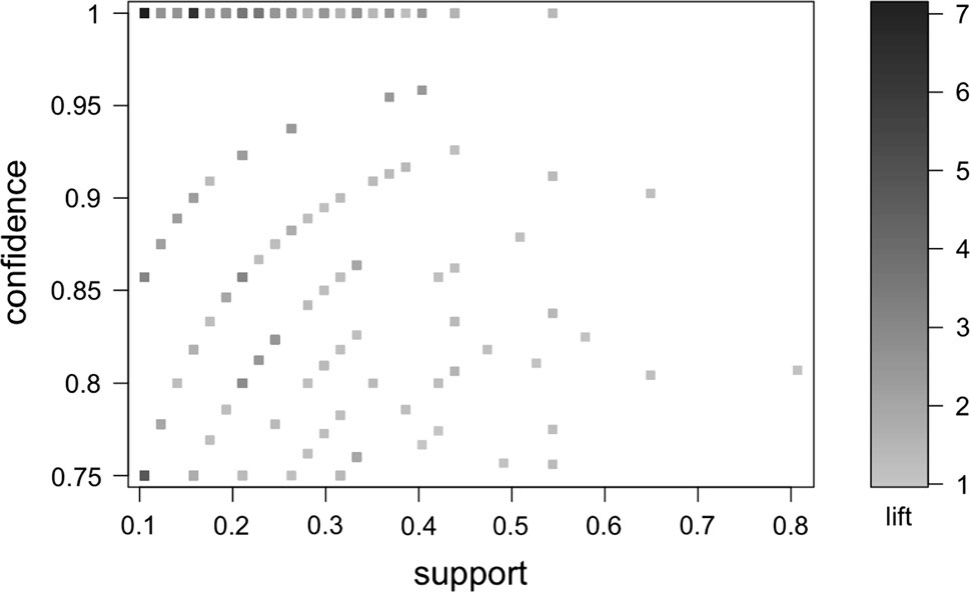
Scatterplot of the confidence, support, and lift of the association rules. I used a 75 % cut-off on the confidence, meaning any rules with confidence less than 75 % are not considered. Support and lift tend to vary across the confidence range; however, there are a respectable number of relatively high confidence and medium support rules. This means that there are very few rules with greater than 75 % confidence that are representative of all chemicals

Across all 3390 rules, there is a wide range of confidence, lift, and support (Fig. 2). Generally, those rules with the highest confidence are the most important ones for this application. Lift is informative as it provides a relative measure of how predictive the rule is compared to random chance. Rules with a lift of 1 are not predicting much better than random. Support is not as useful in this context, as it measures the number of times the rule occurs in the entire database. It is possible that some of our most useful rules are those that occur relatively infrequently within the database. Those structures that are more likely to occur in the database are also those that are the least likely to contain a high amount of information overall. This is similar to the concept that if you are looking for words that best represent a body of work, you are not likely to use the most frequent words, as those are likely to be low information words such as “the”, “a”, and “an”.

The Apriori algorithm was not able to identify any rules where CAT1 or CAT4 was the only element on the right hand side with a confidence greater than or equal to 75 %. Thus, it will not be possible for the current model to assess if a chemical is within CAT1 or CAT4. This represents a data gap; however, this may also reflect the fact that this chemical space does adequately represent acute fish LC50s in the less than 1 mg/L range (CAT1) or the greater than 100 mg/L range (CAT4).

Apriori identified two rules for CAT2 (where CAT2 was the only element on the right hand side) and 27 rules for CAT3 (where CAT3 was the only element on the right hand side) with confidence greater than or equal to 75 %. Thus, these are the only categories where toxicophore identification and chemical activity prediction are likely to be successful.

The analysis/model provides the confidence, or probability, that a combination of structural characteristics is associated with a particular GHS category. Tables of structural alerts based on the chemical structures in the rules and their association with a particular GHS category were able to be built. In addition, the model can be queried with a structure to identify the likely GHS category based on structure alone using a combination of the lift and confidence.

Looking at the rules with the largest lift for each category and using those as a starting point identified potential toxicophores. For CAT2 the following rules have the largest lift:

- {53824505984, 69933794432} => {CAT2}
- {54897200256, 53824505984, 69933794432} => {CAT2}

Since atom-pairs 53824505984 and 69933794432 occur in both rules, and the larger rule does not have any different support, confidence or lift, it is likely that 54897200256 does not add any value to the potential toxicophore. In other words, since (1) the support, confidence and lift for the two rules is the same, and (2) the longer rule contains the same substructures as the shorter rule, except it also includes substructure 54897200256, then it is clear that substructure 54897200256 is adding no additional value. Thus, the toxicophore analysis will center only on atom-pairs 53824505984 and 69933794432 (note that different compounds are used as not all atom-pairs are represented in all compounds).

**Fig. 3:**
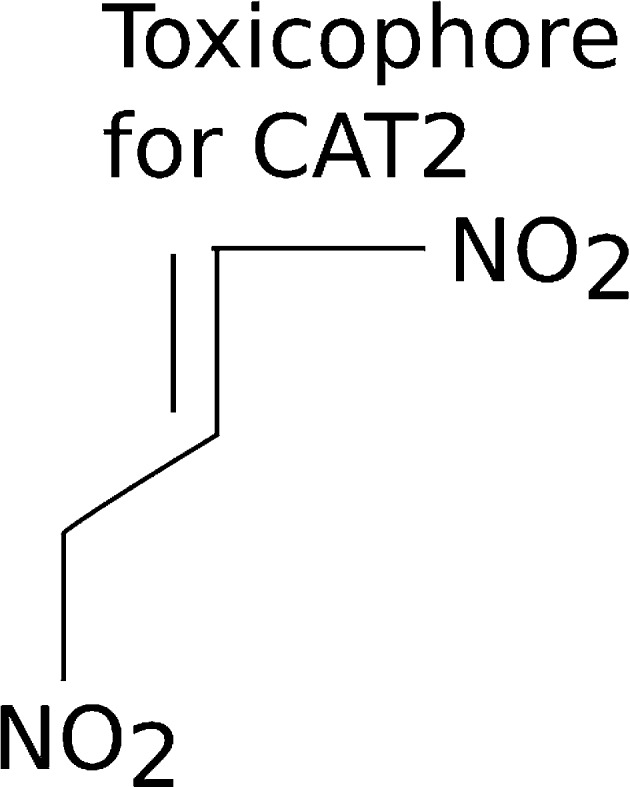
Potential toxicophore for GHS category 2 acute fish toxicity. Based on the chemistry within the dataset, and the highest scoring predictive rule for CAT2, this structure represents a likely toxicophore that manufacturers may want to avoid

These two rules tell state that chemicals with atom pairs consisting of (atom-pair 1, #53824505984) a carbon with 2 neighbors and 1 pi electron that is 3 atoms away from another carbon that also has 2 neighbors and 1 pi electron, and (atom-pair 2, #69933794432) an oxygen with 1 neighbor and 1 pi electron that is 6 atoms away from another oxygen with 1 neighbor and 1 pi electron are likely to predict a chemical to be in CAT2 (Fig. 3).

This toxicophore has a confidence of 75 %, meaning it occurs in 75 % of the chemicals in CAT2. It has a support of 11 %, which means 11 % of all of the chemicals in the database are in CAT2 and have this toxicophore. It has a lift of 2.85, meaning that this rule occurs 2.85× more than would be expected due to chance. Another way to think of lift is that the support for the rule (11 %) is 2.85× greater than the number of chemicals in CAT2 (support for CAT2) times the number of chemicals with this toxicophore (support for the toxicophore).

A similar analysis was performed for CAT3. In this case, the analysis returned one top rule, and that is {54897200256, 53822408832, 53823457408, 54896151680} => {CAT3}. These atom-pairs are: C [3 neighbor(s),1 pi electrons] < —2— > C [3 neighbor(s),1 pi electrons], C [2 neighbor(s),1 pi electrons] < —1— > C [2 neighbor(s),1 pi electrons], C [2 neighbor(s),1 pi electrons] < —2— > C [2 neighbor(s),1 pi electrons], C [3 neighbor(s),1 pi electrons] < —1— > C [3 neighbor(s),1 pi electrons].

**Fig. 4:**
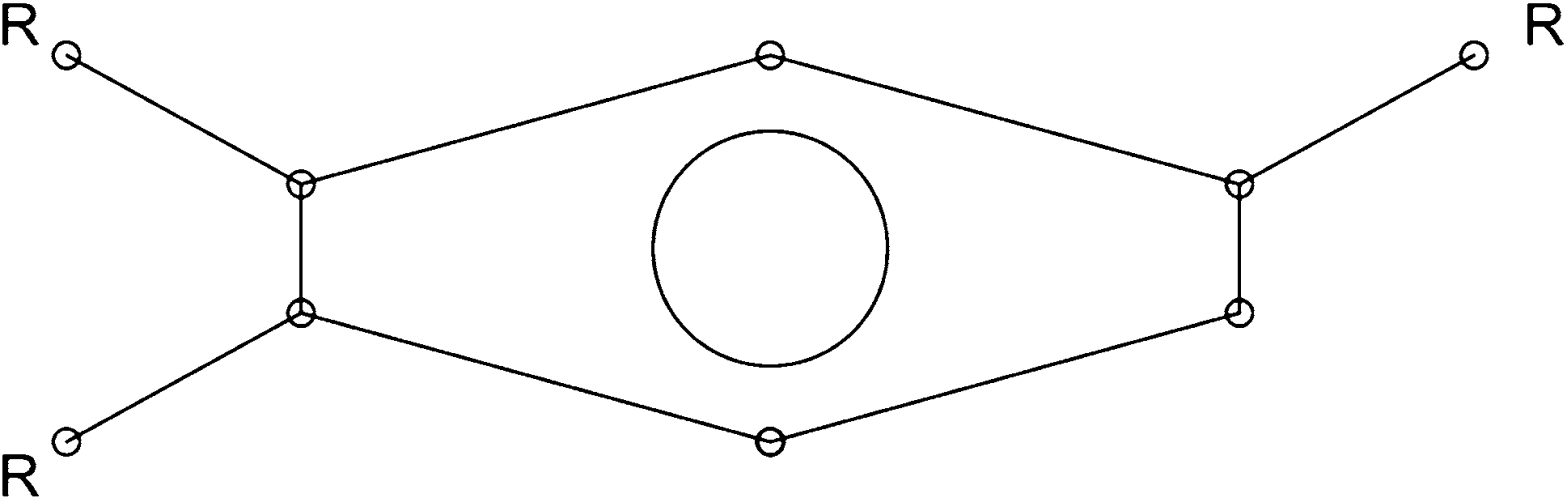
Potential toxicophore for GHS category 3 acute fish toxicity. Based on the chemistry within the dataset, and the highest scoring predictive rule for CAT3, this structure represents a likely toxicophore that may be safer than the one identified for CAT2

The toxicophore predicted for CAT3, based on the chemistry within our dataset, is an aromatic ring with three R-groups, two being situated next to each other on the ring, and the other two carbons away from a carbon attached to one R-group (Fig. 4).

This toxicophore has a confidence of 78 %, meaning it occurs in 78 % of the chemicals in CAT3. It has a support of 25 %, which means 25 % of all of the chemicals in the database are in CAT3 and have this toxicophore. It has a lift of 1.34, meaning that this rule occurs 1.34× more than would be expected due to chance. Another way to think of lift is that the support for the rule (25 %) is 1.34× greater than the number of chemicals in CAT3 (support for CAT3) times the number of chemicals with this toxicophore (support for the toxicophore).

The final examination of this analysis is to examine the model’s ability to predict a chemical’s GHS category. The predicted GHS category was compared to the GHS category assigned based on the acute fathead minnow LC50 data (i.e., ground truth). Only the highest confidence rules for the predictions were used (note, this means that only CAT2 and CAT3 were predicted since CAT1 and CAT4 lack high quality prediction rules). Fivefold cross-validation was performed to assess the model’s ability to accurately predict the GHS category.

**Fig. 5:**
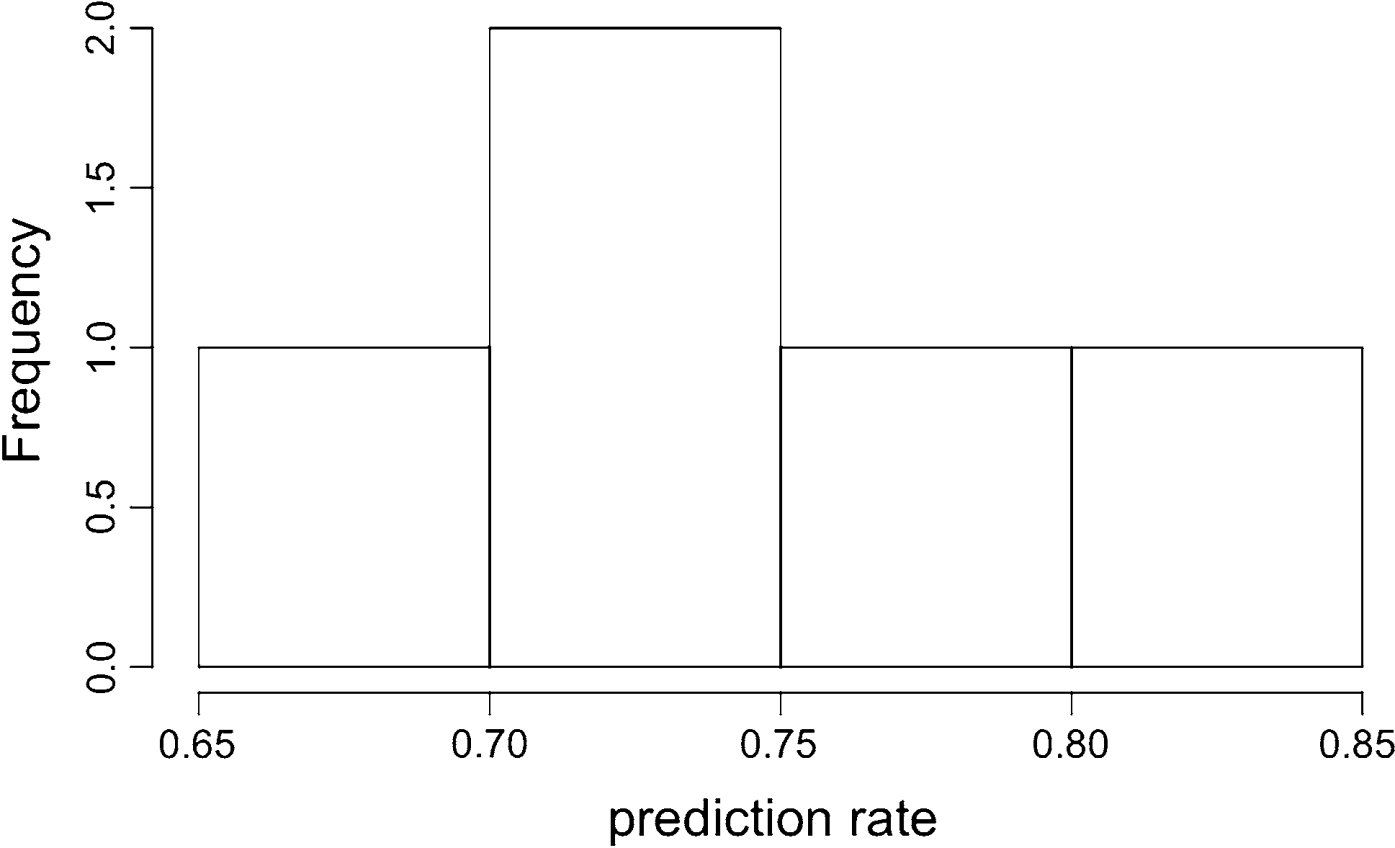
Distribution of prediction rates across the fivefold cross-validation. The average rate across all fivefolds is 74 %

The rate at which chemicals are classified into the GHS categories is approximately 74 %, with a misclassification rate of approximately 26 % (Fig. 5). Given that the chemicals represent four possible classes and the model can only predict two of those, the model does fairly well. However, it should be noted that this is only for those chemicals that the model is capable of classifying.

The model was unable to obtain GHS predictions directly from Apriori for 25/57 of the chemicals. This means that a surrogate approach is required for these remaining chemicals. In a surrogate approach, the chemicals are clustered based on structure and the GHS category predicted by Apriori of the nearest structural neighbor is used.

The surrogate approach was able to assign GHS categories for all of the chemicals lacking a model prediction. This does fairly well overall when predicted categories from the surrogate approach (columns) are compared to ground truth categories (rows; Table 1). There was one misclassification of CAT1 as CAT2, no misclassifications of CAT2, eight misclassifications of CAT3 as CAT2, with five CAT3 correct classifications, and all four CAT4 chemicals were misclassified as CAT2 or CAT3. This is not surprising due to the relatively low numbers of chemicals in CAT1 and CAT4.

**Table 1:** Confusion matrix of GHS category predictions compared to ground truth

	CAT1	CAT2	CAT3	CAT4
CAT1	1	1	0	0
CAT2	0	6	0	0
CAT3	0	8	5	0
CAT4	0	2	2	0

Columns: predicted categories

Rows: ground truth categories

Overall, the modeling and surrogate approaches appear to work reasonably well. Considering the alternative of having no information available at all, or waiting on acute fish studies, the modeling and surrogate approaches will allow for some early decisions to be made. This may help manufacturers identify potentially promising chemicals, and move them through the manufacturing process, or to divest from other emerging products prior to large-scale production. In addition, the modeling approach was able to identify two toxicophores that tend to be associated with CAT2 and CAT3. At present, the dataset is small, and thus it is recommended that further analysis be conducted to identify if there is increased evidence supporting these toxicophores’ association with acute lethality in the fathead minnow model.
